# Progressive IgA Nephropathy Is Associated With Low Circulating Mannan-Binding Lectin–Associated Serine Protease-3 (MASP-3) and Increased Glomerular Factor H–Related Protein-5 (FHR5) Deposition

**DOI:** 10.1016/j.ekir.2017.11.015

**Published:** 2017-11-29

**Authors:** Nicholas R. Medjeral-Thomas, Anne Troldborg, Nicholas Constantinou, Hannah J. Lomax-Browne, Annette G. Hansen, Michelle Willicombe, Charles D. Pusey, H. Terence Cook, Steffen Thiel, Matthew C. Pickering

**Affiliations:** 1 Centre for Complement and Inflammation Research, Imperial College London, London, UK; 2 Department of Biomedicine, Aarhus University, Aarhus, Denmark; 3 Department of Rheumatology, Aarhus University Hospital, Aarhus, Denmark; 4 Renal and Transplant Centre, Imperial College Healthcare NHS Trust, London, UK; 5 Renal and Vascular Inflammation Section, Imperial College London, London, UK

**Keywords:** complement, IgA nephropathy, lectin, MBL

## Abstract

**Introduction:**

IgA nephropathy (IgAN) is characterized by glomerular deposition of galactose-deficient IgA1 and complement proteins and leads to renal impairment. Complement deposition through the alternative and lectin activation pathways is associated with renal injury.

**Methods:**

To elucidate the contribution of the lectin pathway to IgAN, we measured the 11 plasma lectin pathway components in a well-characterized cohort of patients with IgAN.

**Results:**

M-ficolin, L-ficolin, mannan-binding lectin (MBL)–associated serine protease (MASP)-1 and MBL-associated protein (MAp) 19 were increased, whereas plasma MASP-3 levels were decreased in patients with IgAN compared with healthy controls. Progressive disease was associated with low plasma MASP-3 levels and increased glomerular staining for C3b/iC3b/C3c, C3d, C4d, C5b-9, and factor H–related protein 5 (FHR5). Glomerular FHR5 deposition positively correlated with glomerular C3b/iC3b/C3c, C3d, and C5b-9 deposition, but not with glomerular C4d. These observations, together with the finding that glomerular factor H (fH) deposition was reduced in progressive disease, are consistent with a role for fH deregulation by FHR5 in renal injury in IgAN.

**Conclusion:**

Our data indicate that circulating MASP-3 levels could be used as a biomarker of disease severity in IgAN and that glomerular staining for FHR5 could both indicate alternative complement pathway activation and be a tissue marker of disease severity.

IgA nephropathy (IgAN) is a common glomerular pathology that frequently causes renal failure, especially in young people.^1^, ^2^ IgAN is characterized by glomerular deposits of galactose-deficient IgA1.^3^, ^4^ Although a 4-hit theory is proposed for mesangial IgA deposition,^5^ the mechanisms leading to glomerular injury remain poorly understood. The clinical course of IgAN is heterogeneous: after 20 years of follow-up from renal biopsy, up to 40% of patients will reach end-stage renal disease, but 20% of patients will have preserved renal function.^6^ Our incomplete understanding of IgAN pathogenesis limits the development of biomarkers allowing the identification of patients who may benefit from immunosuppression and disease-specific therapies.^2^, ^7^

The complement system is an important inflammation-generating arm of the immune system. Complement activation occurs in IgAN.^8^ Colocalization of glomerular complement C3c with IgA is present in 90% of cases.^3^ Serum levels of activated C3^9^ and mesangial C3 deposition^10^ correlate with loss of renal function. The degree of complement regulation is also important. Imbalances in plasma factor H (fH), an essential negative regulator of C3 activation, and factor H–related (FHR) proteins 1 and 5, that deregulate fH, associate with IgAN.^11^, ^12^ Complement activation leads to the accumulation of C3 proteolytic fragments, such as C3dg, within glomeruli.^8^

The lectin pathway pattern-recognition molecules include MBL (mannan-binding lectin), L-ficolin (also called ficolin-2), M-ficolin (ficolin-1), H-ficolin (ficolin-3), collectin liver 1 (CL-L1, also called CL-10), and collectin kidney 1 (CL-K1 or CL-11). Following interaction with ligands that include pathogen and/or damage-associated molecular patterns, the pattern-recognition molecules trigger complement activation through complexed serine proteases: MBL-associated serine protease (MASP)-1, MASP-2, and MASP-3. Pattern-recognition molecules also can bind nonenzymatic subunits: MBL-associated protein (MAp) 19 and MAp44. The pathway generates a C3-convertase, termed C4bC2b.^13^ The C4b is further processed to C4d. The finding of glomerular C4d in the absence of C1q, the activator of the classic pathway of complement, in IgAN is consistent with lectin pathway activation.^14^

IgAN is characterized by disease flares following respiratory or gastrointestinal tract inflammation^15^; both IgA and the lectin complement pathway are important mediators of innate immunity at these sites. IgAN is associated with higher levels of IgA1 with exposed N-acetyl-galactosamine.^5^, ^16^ N-acetyl-galactosamine is a structure that may trigger lectin pathway activation due to interaction of ficolins with patterns of acetyl-groups.^17^ Furthermore, MBL binds polymeric IgA and triggers complement activation *in vitro*.^18^ Both high and very low MBL levels were associated with poor renal outcomes in a Chinese IgAN population.^19^ Roos *et al.*^20^ demonstrated glomerular MBL, L-ficolin, MASP1/3, and C4d deposition in 25% of patients with IgAN, which associated with disease severity. This finding is supported by the association of glomerular C4d deposition with poor prognosis in IgAN.^12^, ^21^

We hypothesized that the lectin pathway contributes to glomerular inflammation and disease severity in IgAN. We examined (i) levels of circulating lectin pathway components; (ii) glomerular complement deposition; and (iii) glomerular fH, FHR1, and FHR5 deposition in IgAN. Using a cohort of patients with IgAN stratified into those with either stable or progressive disease, we identified circulating lectin pathway components, glomerular complement protein deposition, and immunohistologic evidence of fH deregulation that correlated with disease severity.

## Methods

### Study Cohort and Clinical Measurements

We expanded our previously characterized^11^ Causes and Predictors of Outcome in IgA Nephropathy study cohort of patients with biopsy-proven IgAN to 323 patients (Supplementary Figure S1, UK National Research Ethics Service Committee number 14/LO/0155). Progressive disease was defined by at least 1 of the following criteria: (i) end-stage renal disease without histology evidence of a second pathology causing renal impairment; (ii) biopsy evidence of endocapillary hypercellularity, or (iii) cellular and/or fibrocellular crescents; (iv) treatment with immunosuppression for native IgAN; (v) clinical Henoch-Schonlein purpura, unless spontaneous resolution and >20 years of follow-up with “stable” criteria; or (vi) 50% loss of estimated glomerular filtration rate (eGFR) or average annual loss of eGFR of more than 5 ml/min without evidence of a second pathology causing renal impairment. Stable disease was defined as meeting all of the following: (i) urine protein-creatinine ratio less than 100 units or daily proteinuria of less than 1 g/24 hours; (ii) combined Oxford classification^22^ MEST (mesangial hypercellularity [M], endocapillary hypercellularity [E], segmental glomerulosclerosis [S], interstitial fibrosis/tubular atrophy [T]) score of less than 3; and (iii) average annual loss of eGFR of less than 3 ml/min per 1.73 m^2^. The transplantation cohorts have also been characterized.^11^ Control samples were obtained from healthy volunteers. The eGFR was calculated using the Chronic Kidney Disease Epidemiology Collaboration Creatinine Equation.^23^

### Protein Measurements

Levels of MBL,^24^ M-ficolin,^25^ H-ficolin,^26^ CL-L1,^27^ CL-K1,^28^ MASP-1,^29^ MASP-2,^30^ MASP-3,^31^ MAp19,^32^ and MAp44^31^ were measured using time-resolved immunofluorometric sandwich-type immunoassays as previously described using “in-house” antibodies. Plasma L-ficolin was measured by enzyme-linked immunosorbent assay (Hycult Biotech, Uden, The Netherlands). Serum IgA and galactose-deficient IgA1 levels were measured by enzyme-linked immunosorbent assay.^33^

### Histology

Immunohistochemistry protocols were optimized (Supplementary Figures S2–S4) for formalin-fixed paraffin-embedded renal biopsy tissue with the following antibodies: rabbit polyclonal anti-human C3c (Dako, Glostrup, Denmark), rabbit polyclonal anti-human C4d (DB Biotech, Kosice, Slovakia), mouse monoclonal anti-human factor H (OX-24; Abcam, Cambridge, UK), rabbit polyclonal anti-human C3d (Abcam), mouse monoclonal anti-human C5b9 (Dako), mouse monoclonal anti-human FHR1 (Abnova, Taipei, Taiwan), and rabbit polyclonal anti-human FHR5 (Abnova). The anti-C3c antibody cannot distinguish among C3c, C3b, and iC3b, so we refer to this staining as anti-C3b/iC3b/C3c. We graded antigen-staining intensities from anonymized sections as 0 (absent), 0.5 (minimal), 1+, 2+, and 3+. Staining described as “positive” includes 1+, 2+, and 3+. Staining described as “negative” includes 0 and 0.5. For tubular cell FHR1 staining, we used the area of most intense staining to grade tubular cell FHR1 staining intensity from 0 to 3. We identified 41 IgAN biopsies with median of 6 glomeruli per section (range 2–16). All biopsies had absent or nonsignificant C1q staining documented in clinical reports. We excluded sections that contained <2 nonsclerosed glomeruli.

### Statistical Analysis

Analyses were performed using GraphPad Version 6.00 for Windows (La Jolla, CA). Normally distributed continuous variables were compared using unpaired or paired *t*-test and 1-way analysis of variance for multiple groups. Continuous variables with skewed distribution were tested using Mann-Whitney *U* tests, Kruskal-Wallis tests for multiple groups, and Wilcoxon matched-pairs signed rank test for matched transplant samples. Confidence intervals (CIs) were calculated using the Hodges-Lehmann method; categorical data compared using the Fisher exact test; and correlation assessed using Pearson or Spearman rank tests. We adjusted for multiple analyses with the 2-stage linear step-up procedure of Benjamini, Krieger, and Yekutieli.^34^

## Results

### Plasma M-Ficolin, L-Ficolin, MASP-1, and MAp19 Are Increased, Whereas Plasma MASP-3 Levels Are Reduced in IgAN

Due to the large number of cases and lectin proteins, we quantified lectin pathway plasma concentrations in 2 stages. In the assessment cohort of 125 patients with IgAN and 211 controls, we measured the plasma concentrations of MBL, MASP-1, MASP-2, MASP-3, MAp19, MAp44, CL-K1, CL-L1, M-ficolin, H-ficolin, and L-ficolin (Table 1). We found plasma levels of M-ficolin, L-ficolin, MASP-1, and MAp19 were increased, whereas MASP-3 levels were reduced in patients. This was confirmed in our entire patient cohort (*n* = 323, Table 1). The difference in MBL level seen in the assessment cohort between white patients with IgAN and healthy controls was not replicated. The proportion of MBL-deficient individuals (plasma concentration <100 ng/ml) did not differ between patients and controls (Table 1). Notably, as the 2 proteins are alternative splice products from the same gene (*MASP-2*), 1 patient had a very low plasma MAp19 level (<65 ng/ml) but normal plasma MASP-2 level (180 ng/ml). M-ficolin is expressed in peripheral blood leucocytes.^35^ There was a positive correlation between plasma M-ficolin and white cell count (*r* = 0.38, *P* < 0.0001; Supplementary Figure S5). However, 93% of our cohort had a white cell count in the normal range. L-ficolin, MASP-1, MAp19, and MASP-3 are expressed by hepatocytes.^13^ We identified a positive correlation between plasma MASP-3 and alanine aminotransferase (*r* = 0.31, *P* = 0.0015; Supplementary Figure S5), a marker of liver inflammation. No associations were identified between alanine aminotransferase and plasma L-ficolin, MASP-1, or MAp19 concentrations (data not shown). Plasma MASP-3 levels did not correlate with proteinuria (Supplementary Figure S5).

**Table 1:** Circulating lectin pathway protein levels in IgA nephropathy

Results	Assessment cohort				Complete cohort			
	Patients with IgAN, median (range), *n* = 125	Healthy controls, median (range), *n* = 211	Difference between medians	95% CI	Patients with IgAN, median (range), *n* = 323	Healthy controls, median (range), *n* = 262	Difference between medians	95% CI
MBL, ng/ml	1086 (<10–7202)	1839 (<10–7202)	−753	−587 to 17	1507 (<10–7002)	1557 (<10–7202)	−50	−249 to 145
MBL, white only, ng/ml	1062 (<10–6005), *n* = 85	1839 (<10–7202), *n* = 211	−777^a^	−714 to −9	1471 (<10–6556), *n* = 270	1534 (<10–7202), *n* = 258	−63	−235 to 170
MBL <100, ng/ml	15 (12.0%)	26 (12.3%)			43 (13.3%)	32 (12.2%)		
M-ficolin, ng/ml	4570 (1443–18538)	4201 (1458–10243)	369^b^	181 to 702	5422 (1002–18538)	4124 (1159–10243)	1299^c^	1066 to 1546
H-ficolin, ng/ml	35466 (9617–82262)	36303 (6928–74806)	−837	−3881 to 1185				
L-ficolin, ng/ml	3082 (963–8500)	2751 (982–8267)	341^a^	49 to 576	3463 (872–9230)	2740 (659–8381)	723^c^	543 to 960
CL-L1, ng/ml	535 (295–969)	534 (337–789)	1	−22 to 15				
CL-K1, ng/ml	405 (111–1926)	391 (206–555)	14	−1 to 27				
MASP-1, ng/ml	10325 (4343–20322)	7789 (3188–15494)	2536^c^	1949 to 2936	10323 (2349–56002)	8091 (4490–18130)	2233^c^	1246 to 2327
MASP-2, ng/ml	514 (114–1376)	490 (110–1698)	24	−20 to 69				
MASP-3, ng/ml	6248 (2924–12101)	7038 (2942–14922)	−790^b^	−1092 to −218	5836 (2856–12101)	7028 (2942–14922)	−1192^c^	−1411 to −825
MAp19, ng/ml	588 (<60–928)	489 (186–1140)	99^c^	73 to 125	552 (<60–996)	485 (186–1140)	67^c^	51 to 89
MAp44, ng/ml	2408 (1022–4355)	2351 (1323–4417)	57	−80 to 174				

CI, confidence interval; CL-K1, collectin kidney-1; CL-L1, collectin liver-1; IgAN, IgA nephropathy; MAp, MBL-associated protein; MASP, MBL-associated serine protease; MBL, mannan-binding lectin.

a *P* < 0.05.

b *P* < 0.005.

c *P* < 0.0001.

### Plasma M-Ficolin and MAp19 Levels Are Influenced by Glomerular Filtration Rate

**Figure 1:**
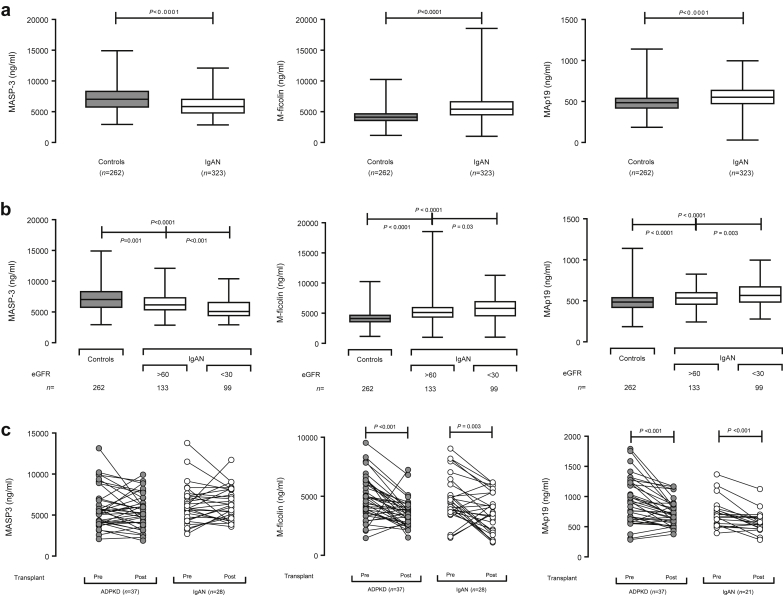
Plasma lectin pathway components in IgA nephropathy. (a) Plasma mannan-binding lectin (MBL)–associated serine protease (MASP)-3 (left), M-ficolin (middle), and MBL-associated protein (MAp) 19 (right) in patients with IgAN and healthy controls. *P* values derived from the Mann-Whitney test. (b) Plasma MASP-3 (left), M-ficolin (middle), and MAp19 (right) in patients with IgAN with either preserved (>60 ml/min) or reduced (<30 ml/min) estimated glomerular filtration rate (eGFR) and healthy controls. *P* values derived from the Kruskal-Wallis test. (c) Plasma MASP-3 (left), M-ficolin (middle), and MAp19 (right) before and after renal transplantation in patients with either IgAN (white circles) or autosomal dominant polycystic kidney disease (ADPKD, gray circles). *P* values derived from Wilcoxon matched-pairs signed rank test.

There was no relationship between eGFR and plasma levels of either L-ficolin or MASP-1 (Supplementary Figure S6). Plasma MASP-3 levels positively correlated (*r* = 0.29, *P* < 0.0001), whereas M-ficolin and MAp19 negatively correlated with eGFR (*r* = −0.17, *P* = 0.002 and *r* = −0.13, *P* = 0.02, respectively; Supplementary Figure S6). Increased plasma M-ficolin and MAp19, and reduced MASP-3 levels, were still evident when we compared patients with preserved eGFR (>60 ml/min) with healthy controls (Figure 1b). These differences increased in magnitude when we compared patients with reduced eGFR (<30 ml/min) with healthy controls (Figure 1b). To determine if these changes were solely due to their association with progressive disease, we compared plasma levels before and after renal transplantation.^11^ Although plasma M-ficolin and MAp19 levels fell significantly posttransplantation in both IgAN and a cohort of adult polycystic kidney disease, plasma MASP-3 levels did not change (Figure 1c). We concluded that M-ficolin and MAp19 levels were influenced by both IgAN and eGFR, whereas MASP-3 levels were influenced only by IgAN.

### MASP-3 Plasma Levels Are Associated With IgAN Severity

**Figure 2:**
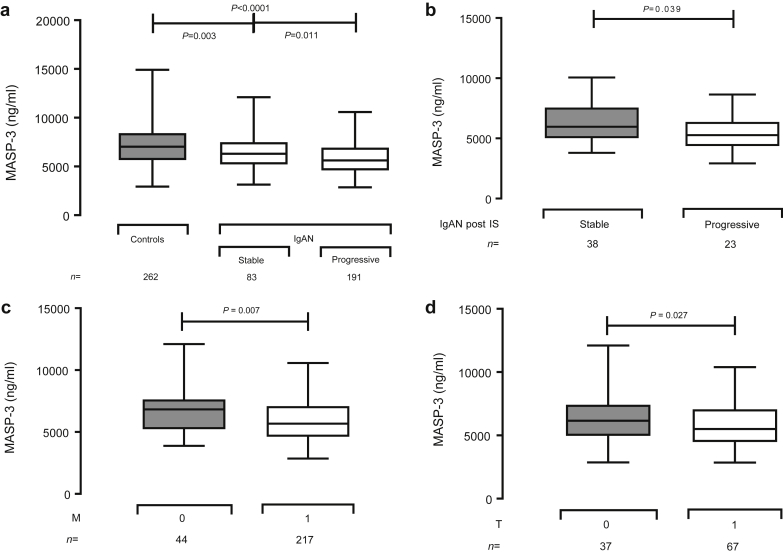
Plasma MASP-3 and progressive IgA nephropathy (IgAN). (a) Plasma mannan-binding lectin (MBL)–associated serine protease (MASP)-3 levels in patients with stable or progressive IgAN compared with healthy controls. *P* values derived from the Kruskal-Wallis test. (b) Plasma MASP-3 levels in patients with stable or progressive IgAN despite immunosuppressive therapy (IS). *P* values derived from the Mann-Whitney test. (c) Plasma MASP-3 plasma levels in patients with IgAN without (0, gray box) and with (1, white box) mesangial hypercellularity (M). (d) Plasma MASP-3 plasma levels in patients with IgAN without (0, gray box) and with (1, white box) tubular atrophy. *P* values derived from the Mann-Whitney test.

To explore the significance of the altered lectin pathway levels in IgAN, we compared patients with either stable or progressive disease. M-ficolin, L-ficolin, MASP-1, and MAp19 did not differ between the groups (Supplementary Figure S7); however, MASP-3 levels were reduced in patients with progressive disease (Figure 2a), including those with progressive disease despite immunosuppressive therapy (Figure 2b). Given the demonstrated stability of MASP-3 serology levels over time,^31^, ^36^ we compared plasma levels (sampled at recruitment) with the Oxford Classification of IgA Nephropathy^22^ scores from diagnostic renal biopsies. Plasma MASP-3 levels were lower in the patient cohort with biopsy evidence of mesangial hypercellularity (Figure 2c) and tubular atrophy (Figure 2d). Plasma MAp19 levels were higher in patients with segmental sclerosis (mean 576 vs. 539 ng/ml; difference 37; 95% CI: 2–73 ng/ml; *P* = 0.040). We did not identify associations between histology parameters and M-ficolin, L-ficolin, or MASP-1 levels.

### Glomerular Complement Deposition Is Associated With Progressive IgAN

**Figure 3:**
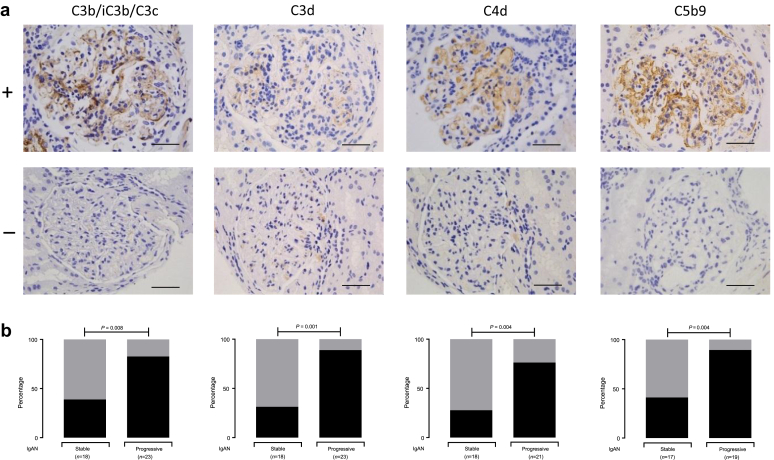
Glomerular complement staining in IgA nephropathy (IgAN). (a) Representative images for complement C3b/iC3b/C3c, C3d, C4d, or C5b9. The top row represents present (+) and the bottom row represents negative (−) staining. Original magnification ×400. Bar = 100 μm. (b) Proportion of cases with present (black) versus absent/uncertain (gray) glomerular staining in stable and progressive IgAN. *P* values derived from the Fisher exact test.

To understand the significance of the association between MASP-3 levels and progressive IgAN, we assessed complement deposition in our stable and progressive cohorts (Figure 3). In progressive compared with stable disease, there was proportionately greater glomerular staining for C3b/iC3b/C3c (odds ratio [OR]: 5.66; 95% CI: 1.49–23.39; *P* = 0.02), C3d (OR: 17.6; 95% CI: 3.01–89.97; *P* = 0.001), C4d (OR: 8.32; 95% CI: 2.00–30.33; *P* = 0.004), and C5b9 (OR: 12.14; 95% CI: 1.95–61.35; *P* = 0.004). Glomerular C5b-9 staining, a marker of complement terminal pathway activation, significantly correlated with both glomerular C3b/iC3b/C3c and C3d but not C4d staining (Table 2). There was no correlation between glomerular C4d and either C3d or C3b/iC3b/C3c.

**Table 2:** Correlation of mesangial complement antigen intensity in IgA nephropathy native renal biopsies

Correlation coefficient *R* values	C3b/iC3b/C3c	C3d	C4d	C5b9	fH	FHR1	FHR5
**C3b/iC3b/C3c**	–	0.45 (*P* = 0.01)	0.31 (*P* = 0.09)	0.57 (*P* < 0.001)	0.06 (*P* = 0.52)	−0.28 (*P* = 0.8)	0.64 (*P* < 0.001)
**C3d**		–	0.22 (*P* = 0.20)	0.53 (*P* = 0.003)	−0.29 (*P* = 0.11)	−0.11 (*P* = 0.42)	0.68 (*P* < 0.001)
**C4d**			–	0.34 (*P* = 0.08)	−0.22 (*P* = 0.19)	0.08 (*P* = 0.45)	0.27 (*P* = 0.12)
**C5b9**				–	−0.26 (*P* = 0.14)	−0.31 (*P* = 0.09)	0.75 (*P* < 0.001)
**fH**					–	−0.33 (*P* = 0.09)	−0.16 (*P* = 0.30)
**FHR1**						–	−0.31 (*P* = 0.09)
**FHR5**							–

A correlation of complement antigen-staining intensities in sections from the same biopsy, using the 0, 0.5, 1+, 2+, and 3+ scale. *R* values are calculated from Spearman’s rank correlation. *P* values shown have been adjusted for multiple analyses to minimize the false discovery rate (using the 2-stage linear step-up procedure of Benjamini, Krieger, and Yekutieli). fH, factor H; FHR1, factor H–related protein 1; FHR5, factor H–related protein 5.

### Glomerular FHR5 Deposition Is Associated With Progressive IgAN

**Figure 4:**
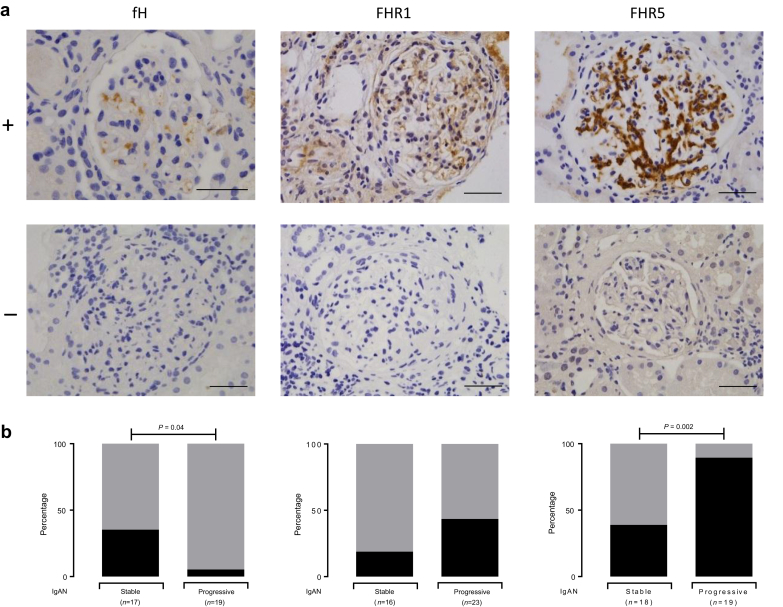
Glomerular staining for alternative pathway regulators in IgA nephropathy (IgAN). (a) Representative images for complement factor H (fH), and factor H–related proteins 1 (FHR1) and 5 (FHR5). The top row represents positive staining (+) and the bottom row represents negative staining (−). Original magnification ×400. Bar = 100 μm. (b) The proportion of cases with positive (black) versus absent/minimal (gray) glomerular staining in stable and progressive IgAN. *P* values derived from the Fisher exact test.

**Figure 5:**
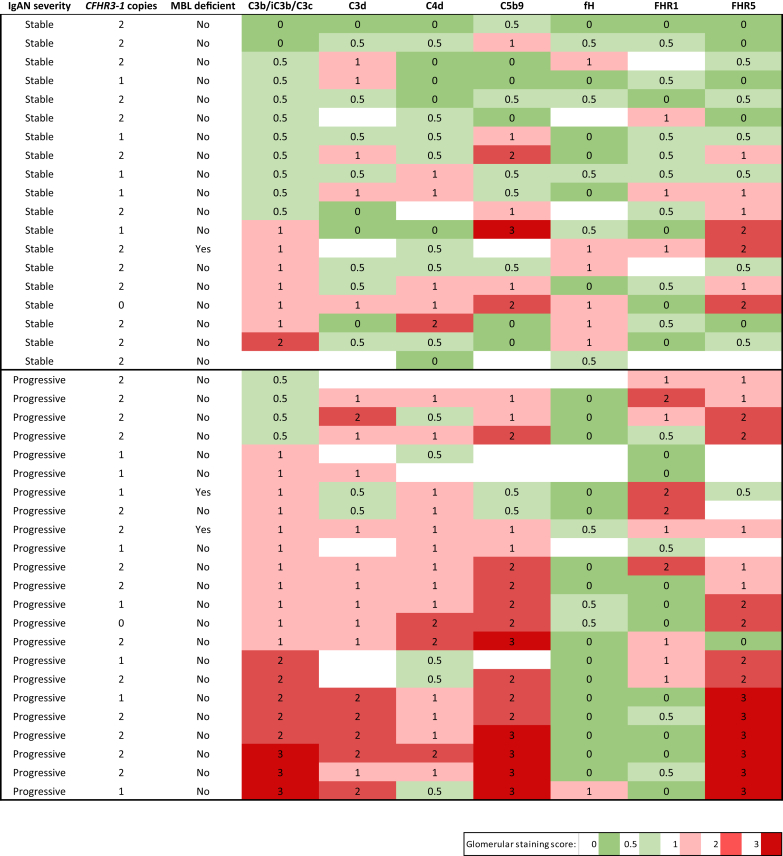
Complement glomerular deposition in IgA nephropathy (IgAN). Glomerular staining intensity scores from surplus native renal biopsy tissue from patients with either stable or progressive IgAN. Each row represents information from a single patient. Staining intensity was scored: 0, absent; 0.5, minimal; 1, mild; 2, moderate; 3, strong. Filled cells indicate insufficient renal tissue to perform staining. Mannan-binding lectin (MBL) deficiency was defined as a plasma level of less than 100 ng/ml. *CFHR3-1*, complement factor H–related 3 and 1 genes; fH, complement factor H; FHR1, factor H–related protein 1; FHR5, factor H–related protein 5.

We have previously shown levels of negative (fH) and positive regulators (FHR1, FHR5) of the complement alternative pathway associated with progressive IgAN.^11^ In progressive disease, there was more glomerular staining for FHR5 (OR: 13.4; 95% CI: 2.2–66.9; *P* = 0.002) and a trend for greater FHR1 staining (Figure 4). In contrast, glomerular staining for fH was significantly reduced in progressive compared with stable disease (OR: 0.10; 95% CI: 0.008–0.87; *P* = 0.04). Glomerular FHR5 staining correlated with glomerular C3b/iC3b/C3c, C3d, and C5b-9 but not C4d (Table 2). In aggregate, a heat map of the glomerular staining data showed that the renal biopsies from patients with progressive disease had more staining for C3b/iC3b/C3c, C3d, C4d, C5b9, and FHR5 than those with stable disease (Figure 5). Interestingly, the combination of FHR5 staining with negative fH staining was significantly more common in patients with progressive (15/18, 88.2%) versus stable (4/16, 25%) disease (OR: 15; 95% CI: 2.5–62.6; *P* = 0.001). Only 1 of 14 (6.7%) stable patients had glomerular FHR1 staining and negative fH staining compared with 9 of 19 (47.4%) in the progressive cohort (OR: 12.6; 95% CI: 1.7–146.4; *P* = 0.02). Surplus renal biopsy tissue was available from 1 patient with stable and 1 with progressive IgAN who had homozygous deletion of the CFHR3 and 1 genes. Glomerular FHR1 staining was negative in both cases, but glomerular C3b/iC3b/C3c, C3d, C4d, C5b9, and FHR5 was detectable (Figure 5). Surplus renal biopsy tissue was available from 3 patients with MBL deficiency: 1 with stable and 2 with progressive disease (Figure 5). Glomerular C4d deposition was present in the 2 patients with progressive disease but negative in the patient with stable disease. Glomerular C3b/iC3b/C3c was detectable in all 3.

The IgAN cohort demonstrated a range of tubular FHR1 staining prevalence and intensity (Supplementary Figure S8). FHR1 staining was often present and absent in adjacent tubule segments (Supplementary Figure S8). Eleven patients showed no tubular staining for FHR1, 7 had 1+, 19 had 2+, and 4 had tubular cell FHR1 staining of 3+ intensity. The intensity of tubular cell FHR1 staining did not correlate with proteinuria, glomerular filtration, or the presence of tubular atrophy at the time of biopsy. And there was no correlation with disease severity. We did not have access to stored plasma samples to quantify the circulating FHR1 concentration at the time of biopsy.

## Discussion

We observed increased circulating M-ficolin, L-ficolin, MASP-1, and MAp19 levels in patients with IgAN. Conversely MASP-3 levels were reduced and associated with progressive disease. Renal impairment partially explained the differences in M-ficolin and MAp19 levels, because these levels changed significantly following renal transplantation. M-ficolin is mainly synthesized in monocytes and granulocytes,^37^ but almost all of our patients had a normal white cell count. Although MASP-3 showed a positive correlation with alanine aminotransferase, we identified negative correlations with MASP-3 levels and IgAN severity. We therefore think it is unlikely that the M-ficolin and MASP-3 results are explained by confounding with leucocyte count and liver inflammation, respectively. Furthermore, MBL,^38^ M-ficolin,^25^ L-ficolin,^39^ MASP-1,^40^ and MAp19^32^ are not acute-phase proteins. Circulating MASP-3 levels did not correlate with proteinuria, excluding this as a cause for the low levels, and there is no clear genotype-phenotype correlation to explain the variation in MASP-3 levels.^41^

How these changes relate to IgAN pathogenesis is unclear. Increased MASP-1^42^ and MAp19^42^ levels occur in lupus nephritis, so these changes are not specific to IgAN. Glomerular L-ficolin and MASP1/3 staining correlated with IgAN severity,^20^ but circulating L-ficolin levels did not.^20^ In fact, reduced circulating L-ficolin levels have been reported in lupus nephritis,^43^ although this was not replicated in a Japanese cohort,^44^ in addition to reduced M-ficolin levels.^29^ M-ficolin^35^ and L-ficolin^45^ are capable of triggering lectin pathway activation, and MASP-1 is essential for MASP-2 and lectin complement activation,^46^ so higher levels of these 3 components could be associated with greater complement activation within the kidney. The significance of the raised MAp19 is unclear. It has been shown to have a regulatory role on lectin pathway activation *in vitro*,^47^ but the physiological relevance of this is unknown.^32^

MASP-3 levels are lower in patients with systemic lupus erythematosus with nephritis compared with those without,^42^ so low MASP-3 levels are not specific to IgAN. When MASP-3 binds to pattern-recognition molecules, it can displace MASP-2 and MASP-1,^48^ and, because it does not lead to convertase formation,^49^ inhibit activation.^48^, ^50^ Low levels of MASP-3 could be associated with increased complement activation. However, MASP-3 activates pro-factor D to factor D,^51^ a requirement for C3-convertase formation.^52^ Whether or not glomerular activation of the alternative pathway in either lupus nephritis or IgAN directly influences MASP-3 levels is unknown.

We replicated the association between IgAN severity and glomerular C3b/iC3b/C3c^10^ and C4d.^20^, ^21^ Our data also showed an association between progressive IgAN and glomerular C3d and C5b9. Glomerular C4d did not correlate with either C3b/iC3b/C3c or C5b9. This may reflect technical limitations (e.g., sample size, staining procedure), but also could be because the amount of C4d, compared with C3 and C5b-9, will be lower after glomerular complement activation. Previous studies have identified glomerular C3 and C5b9 in most C4d-positive and -negative cases, but did not record antigen correlations.^20^, ^21^

The correlation of glomerular FHR5 deposition with progressive IgAN is a key finding of our study. Glomerular FHR5 correlated with C3b/iC3b/C3c, C3d, and C5b9 staining, as previously reported.^53^ In a proteomic analysis, glomerular FHR5 was 1.79 times more abundant in patients with progressive versus stable IgAN.^54^ FHR5 antagonizes the ability of fH to negatively regulate C3 activation.^55^, ^56^ Consequently, it was interesting that patients with progressive disease had more cases of glomerular FHR5 staining in the absence of fH. Notably, there are phenotypic similarities between IgAN and familial C3 glomerulopathy associated with mutant FHR5 proteins.^57^, ^58^

Considering the genetic and serology associations between IgAN and FHR1,^11^, ^12^, ^59^, ^60^ it was surprising that glomerular FHR1 was absent in more than 50% of progressive IgAN biopsies. Unlike FHR5, glomerular FHR1 did not correlate with other complement antigens. This could be explained by differences in binding avidity of FHR1 and FHR5 to C3b, iC3b, and C3dg.^55^ Nevertheless, our data indicate a more prominent role for FHR5 than FHR1 in complement activation in IgAN. Notably, 1 patient with progressive disease was deficient in FHR1.

The cause of tubular cell FHR1 staining is unclear. It did not correlate with proteinuria at the time of biopsy. It may result from changes to the tubular cell membrane as a consequence of nephron loss or changes in tubular fluid characteristics, such as acidity, or tubular epithelia or glycocalyx features.

Our complement staining data demonstrated the pathogenic heterogeneity of IgAN. For example, the co-deposition of FHR5, C3d, C3b/iC3b/C3c, and C5b9, especially in the absence of fH, implies FHR5-dependent fH deregulation and alternative pathway activation, and glomerular co-deposition of C4d with C3b/iC3b/C3c and C5b9 may reflect complement activation triggered by the lectin pathway. Interestingly, all 4 biopsies from patients with progressive disease and negative glomerular C4d had FHR5 staining. Identifying and understanding this heterogeneity of complement activity might be clinically important because we now have the ability to target complement activation at specific points in the activation sequence. For example, OMS721, a monoclonal antibody targeting MASP-2, was recently designated breakthrough therapy status for IgAN treatment; Eculizumab, a C5 inhibitor, has been used in recurrent^61^ and progressive^62^, ^63^ IgAN; and factor D inhibitors are in clinical trials for C3 glomerulopathy.^64^

**Figure 6:**
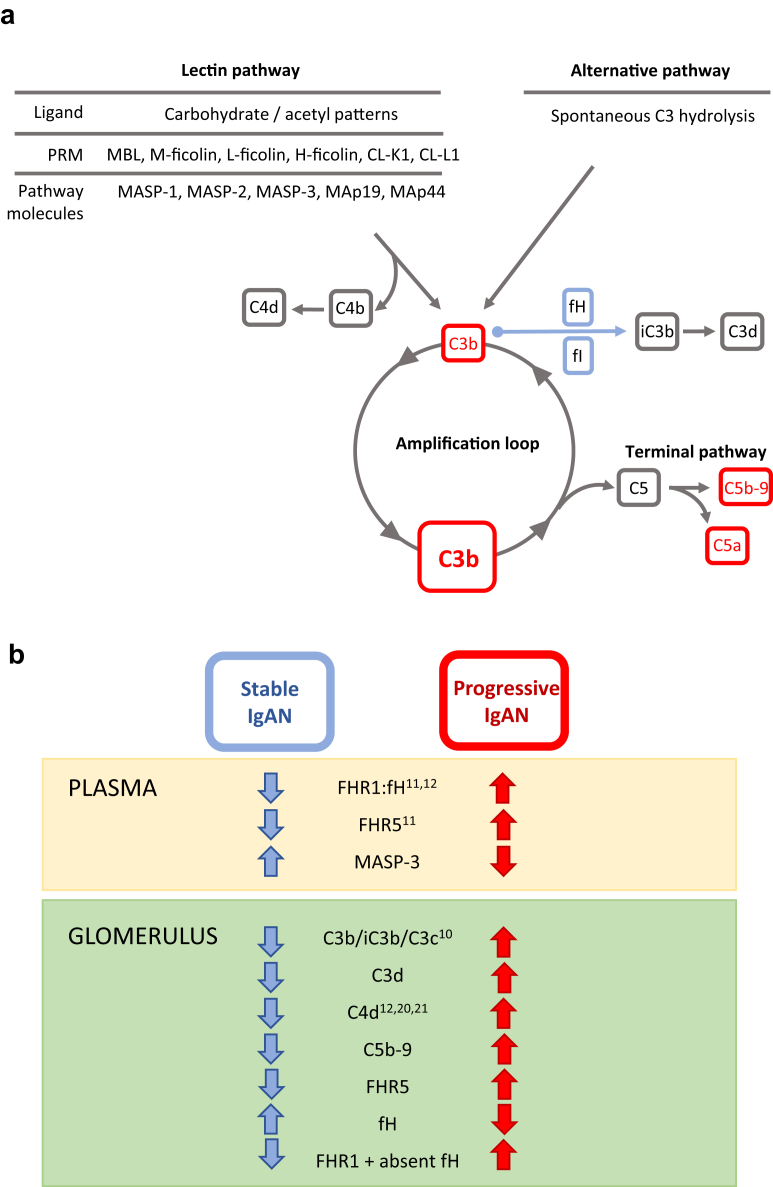

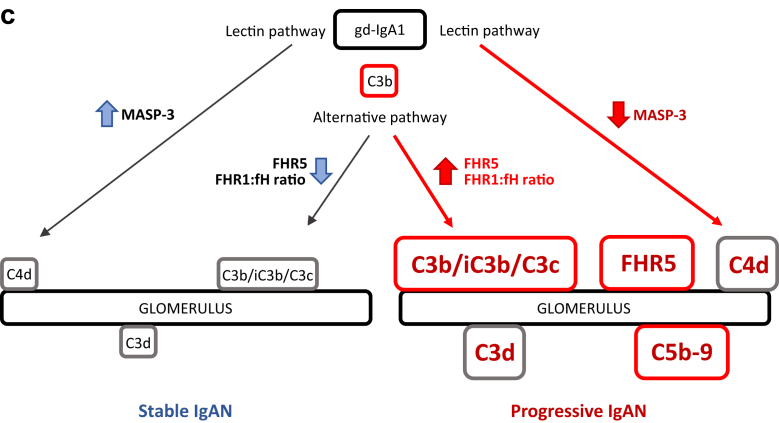
Complement and IgA nephropathy. (a) Schematic diagram depicting lectin and alternative pathway complement activation. Lectin pathway activation is triggered by the binding of pattern-recognition molecules (PRMs) to carbohydrate or acetyl molecular patterns. Alternative pathway activation happens through the spontaneous, constant generation of reactive forms of C3. Both pathways result in the generation of C3b. C3b can be proteolytically cleaved to iC3b and C3d by complement factor I in the presence of cofactors, such as complement factor H. Similarly, C4b produced during lectin pathway activation can be cleaved to C4d. C3b generation can be rapidly amplified through an amplification loop. This results in the generation of large amounts of the opsonin C3b and can trigger complement C5 activation. This leads to the generation of the anaphylatoxin C5a, and the membrane attack complex (C5b-9) through the terminal pathway. fH, factor H; fI, factor I; MAp, MBL-associated protein; MASP, MBL-associated serine protease; MBL, mannose-binding lectin. (b) Complement proteins and severity of IgA nephropathy. Within plasma, increased levels of FHR1,^11^, ^12^ FHR5,^11^ and the FHR1:fH ratio^11^, ^12^ associate with progressive IgAN. Conversely, we found that reduced levels of MASP-3 associated with progressive disease. Within glomeruli, we replicated the association between increased C4d^12^, ^20^, ^21^ and C3b/iC3b/C3c^10^ with IgAN severity, and we showed that increased glomerular C3d, C5b-9, and FHR5 associated with progressive disease. The presence of FHR1 in the absence of fH was also more frequently seen in patients with progressive disease. FHR1, factor H–related protein 1; FHR5, factor H–related protein 5. (c) Hypothetical depiction of glomerular complement activation in IgA nephropathy. Galactose-deficient IgA1 (gd-IgA1) activates the lectin and alternative complement pathways in IgAN.^8^ Glomerular complement deposition is enhanced in progressive disease. Glomerular complement activation is influenced by FHR5 and the FHR1-fH ratio and associated with changes in circulating MASP-3 levels. Changes in FHR1, FHR5, and fH influence complement activation through the alternative pathway. fH negatively regulates activation, whereas FHR1 and FHR5 promote activation through antagonizing fH (“fH de-regulation”). The mechanism through which circulating MASP-3 levels fall in progressive disease are not understood but are presumed to be linked to lectin pathway activation. Red text highlights proteins demonstrated to associate with progressive IgAN and larger boxes indicate more deposition.

Although our observations require confirmation in larger IgAN cohorts, our data indicate (Figure 6) that (i) circulating MASP-3 is a potential biomarker of disease severity in IgAN; and (ii) glomerular FHR5 staining of diagnostic biopsies can identify those with severe disease who are at risk of progression to renal impairment.

## Disclosure

All the authors declared no competing interests.

## Supplementary Material

Figure S1 Table of IgA nephropathy and healthy control cohort characteristics.

Figure S2 Representative images of renal immunohistochemistry staining for complement pathway antigens: C3b/iC3b/C3c, C3d, C4d, and C5b-9. Original magnification ×400. Bar = 100 μm.

Figure S3 Representative images of renal immunohistochemistry staining for complement pathway antigens: FHR1, FHR5, and fH. Original magnification ×400. Bar = 100 μm.

Figure S4 Representative image of liver immunohistochemistry staining for fH. Original magnification ×400. Bar = 100 μm.

Figure S5 Graphs showing the (a) correlation of plasma MASP-3 levels and urine protein-creatinine ratio (PCR) in patients (*n* = 223) with IgA nephropathy (IgAN); (b) the correlation of plasma M-ficolin with white cell count (WCC) in patients (*n* = 108) with IgAN; and (c) the correlation between mannan-binding lectin (MBL)-associated serine protease (MASP)-3 with alanine aminotransferase (ALT) in patients (*n* = 108) with IgAN.

Figure S6 Graphs showing the correlation between estimated glomerular filtration rate (eGFR) and (a) M-ficolin, (b) mannan-binding lectin (MBL)-associated protein (MAp) 19, (c) MBL-associated serine protease (MASP)-3 levels, (d) L-ficolin, and (e) MASP-1 levels.

Figure S7 Table of circulating lectin pathway protein levels in stable and progressive IgA nephropathy.

Figure S8 Renal immunohistochemistry staining for factor H–related protein 1 (FHR1) in IgA nephropathy. Representative images of tubular staining intensity 3+ (a), 2+ (b), 1+ (c), and 0 (d–f). (f) Representative image from a patient with complete FHR1 deficiency. Bar = 100 μm. Correlation of tubular FHR1 staining intensity with estimated glomerular filtration rate (eGFR, g) at the time of biopsy, urine protein-creatinine ratio (UPCR) at the time of biopsy (h), the presence (T1 or T2) or absence (T0) of tubular atrophy from the biopsy report (i); and disease severity at enrollment (j). No correlations reached statistical significance.
