# Supplementary material for: Progressive IgA Nephropathy Is Associated With Low Circulating Mannan-Binding Lectin–Associated Serine Protease-3 (MASP-3) and Increased Glomerular Factor H–Related Protein-5 (FHR5) Deposition
Source: Kidney Int Rep. 2017 Nov 29;3(2):426–38. doi: 10.1016/j.ekir.2017.11.015 (PMC5932138; doi:10.1016/j.ekir.2017.11.015)
Supplement: Figure S1 — Table of IgA nephropathy and healthy control cohort characteristics. [file mmc1.pdf]

| Characteristic | All IgAN (n=323) | Progressive IgAN (n=191) | Stable IgAN (n=83) | Danish Control cohort (n=211) | London Control cohort (n=51) |
|---|---|---|---|---|---|
| Male/Female | 214/109 | 127/63 | 51/33 | 110/101 | 25/26 |
| Caucasian/Non-Caucasian | 271/52 | 155/36 | 73/10 | 211/0 | 47/4 |
| Median age (range) – years | 48 (18-84) | 48 (19-84) | 48 (18-82) | 35 (26-49) | 31 (20-66) |
| Median eGFR – ml/min | 52.6 (28.7-81.8) | 44.2 (17.7-71.3)[1] | 77.3 (47.6-106) | ND | ND |
| Median urine PCR – mg/mmol | 44 (16-117, n=220) | 46 (16-141.5, n=125) | 40 (15-90, n=76) | ND | ND |
| Median anti-hypertensive drug classes per patient | 1.6 | 1.6 | 1.4 | ND | ND |
| Patients with ACEi/ARB, excluding dialysis patients - % | 73.7 (n=199) | 77.3 (n=176) | 79.8 (n=83) | ND | ND |
| Median systolic/diastolic blood pressure – mmHg | 134 (122-145) / 79 (70-88, n=294) | 136 (124 - 146)[2] / 79 (70-88, n=181) | 128 (116 - 140) / 77.5 (70-85, n=83) | ND | ND |
| Median duration biopsy to recruitment (months) | 56.1 (22.8-104.7) | 52.2 (23.3-94.8) | 68.4 (22.8-171.3) | ND | ND |
| Reach ESRF - % | 34.9 | 57.5[3] | 0 | ND | ND |
| History of macroscopic haematuria - % | 27.8 | 20.1[3] | 44.9 | ND | ND |
| Diagnosis of Henoch-Schonlein purpura - % | 6.4 | 8.9[3] | 0 | ND | ND |
| Median serum IgA – g/l | 3.4 (2.9-4, n=293) | 3.3 (2.7-3.8, n=178)[4] | 3.7 (3.2-4.3, n=88) | ND | 2.8 (2.2-3.2, n=57)[5] |
| Median serum gd-IgA1 – AU | 0.50 (0.41-0.58, n=293) | 0.52 (0.42-0.59, n=178)[6] | 0.47 (0.37-0.55, n=88) | ND | 0.42 (0.33-0.55, n=57)[7] |

**Supplemental figure 1.** Cohort characteristics.

Values within parentheses represent interquartile range and number analysed if less than the respective cohort numbers. IgAN – IgA nephropathy; ACEi - Angiotensin-converting enzyme inhibitor; ARB - Angiotensin receptor blocker; AU – arbitrary units; ESRF – End-stage renal disease; gd-IgA1 - galactose-deficient IgA1; PCR – protein:creatinine ratio; ND – Not done. [1]$P$ <0.0001, [2]$P$ =0.0014, [3]$P$ <0.0001 and [4]$P$ =0.0007 vs. stable IgAN; [5]$P$ <0.0001 vs. all IgAN; [6]$P$ =0.01 vs. stable IgAN; [7]$P$ =0.015 vs. all IgAN;
