# Supplementary material for: Progressive IgA Nephropathy Is Associated With Low Circulating Mannan-Binding Lectin–Associated Serine Protease-3 (MASP-3) and Increased Glomerular Factor H–Related Protein-5 (FHR5) Deposition
Source: Kidney Int Rep. 2017 Nov 29;3(2):426–38. doi: 10.1016/j.ekir.2017.11.015 (PMC5932138; doi:10.1016/j.ekir.2017.11.015)
Supplement: Figure S2 — Representative images of renal immunohistochemistry staining for complement pathway antigens: C3b/iC3b/C3c, C3d, C4d, and C5b-9. Original magnification ×400. Bar = 100 μm. [file mmc2.pdf]

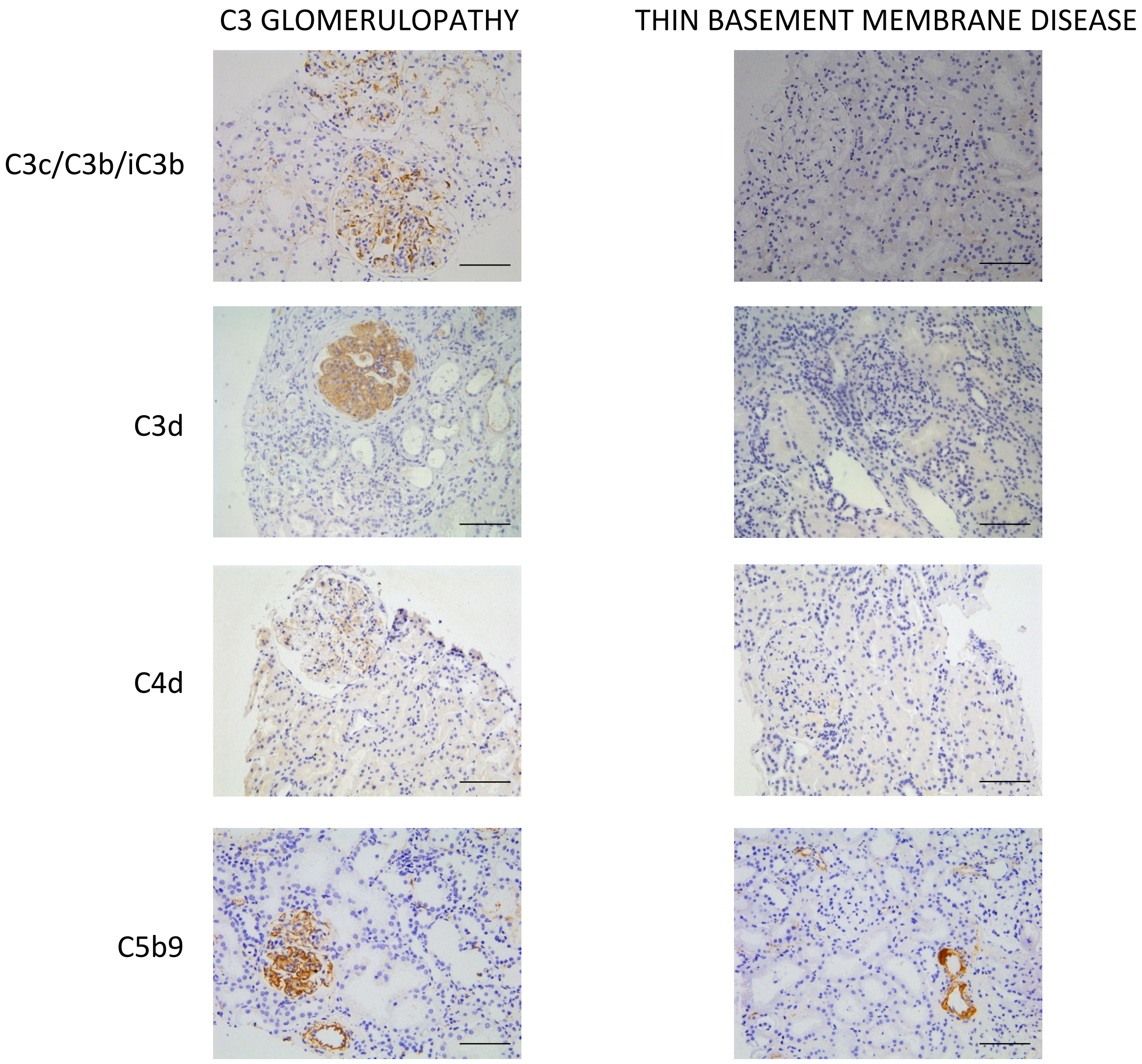

Supplemental figure 2. Representative images of renal immunohistochemistry staining for complement pathway antigens: C3c/C3b/iC3b, C3d, C4d and C5b-9. To optimise our staining protocols we used renal tissue from patients with either C3 glomerulopathy (positive control) or thin basement membrane disease (negative control). Bar represents 100 μm.
