# Supplementary material for: Progressive IgA Nephropathy Is Associated With Low Circulating Mannan-Binding Lectin–Associated Serine Protease-3 (MASP-3) and Increased Glomerular Factor H–Related Protein-5 (FHR5) Deposition
Source: Kidney Int Rep. 2017 Nov 29;3(2):426–38. doi: 10.1016/j.ekir.2017.11.015 (PMC5932138; doi:10.1016/j.ekir.2017.11.015)
Supplement: Figure S3 — Representative images of renal immunohistochemistry staining for complement pathway antigens: FHR1, FHR5, and fH. Original magnification ×400. Bar = 100 μm. [file mmc3.pdf]

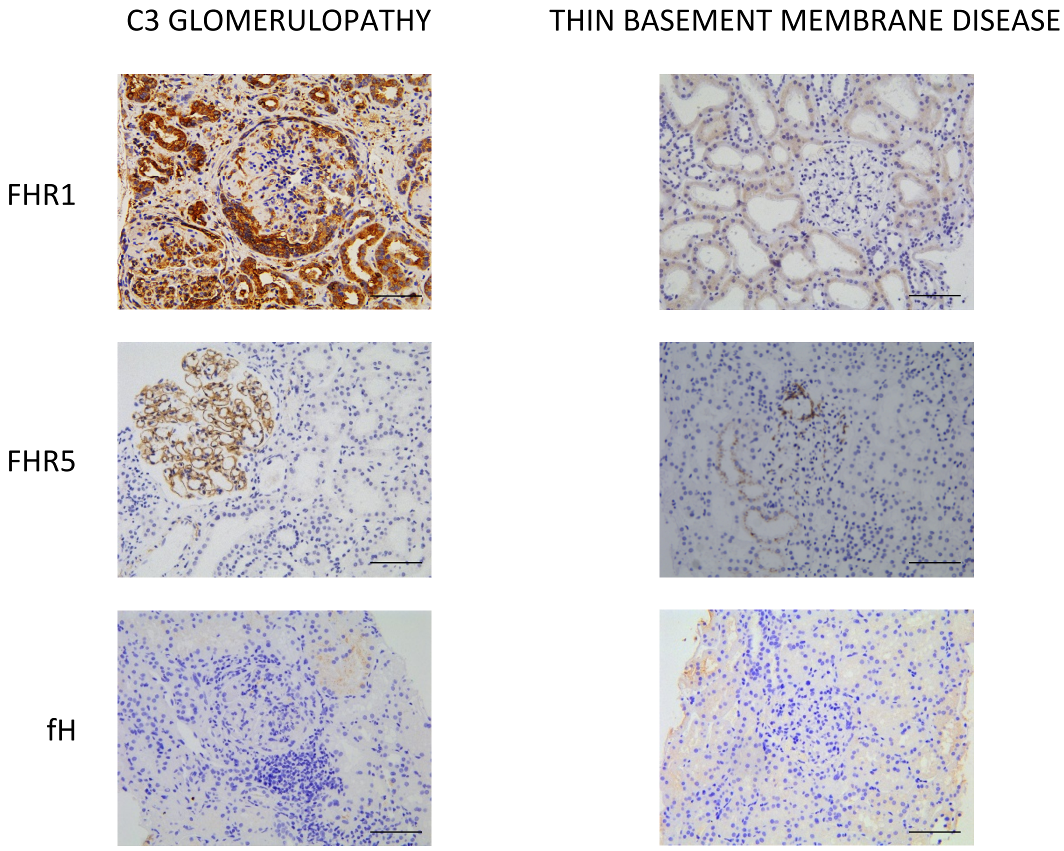

Supplemental figure 3. Representative images of renal immunohistochemistry staining for complement pathway antigens: FHR1, FHR5 and fH. To optimise our staining protocols we used renal tissue from patients with either C3 glomerulopathy (positive control) or thin basement membrane disease (negative control). FHR − factor H-related, fH − factor H. Bar represents 100 µm.
