# Supplementary material for: Progressive IgA Nephropathy Is Associated With Low Circulating Mannan-Binding Lectin–Associated Serine Protease-3 (MASP-3) and Increased Glomerular Factor H–Related Protein-5 (FHR5) Deposition
Source: Kidney Int Rep. 2017 Nov 29;3(2):426–38. doi: 10.1016/j.ekir.2017.11.015 (PMC5932138; doi:10.1016/j.ekir.2017.11.015)
Supplement: Figure S4 — Representative image of liver immunohistochemistry staining for fH. Original magnification ×400. Bar = 100 μm. [file mmc4.pdf]

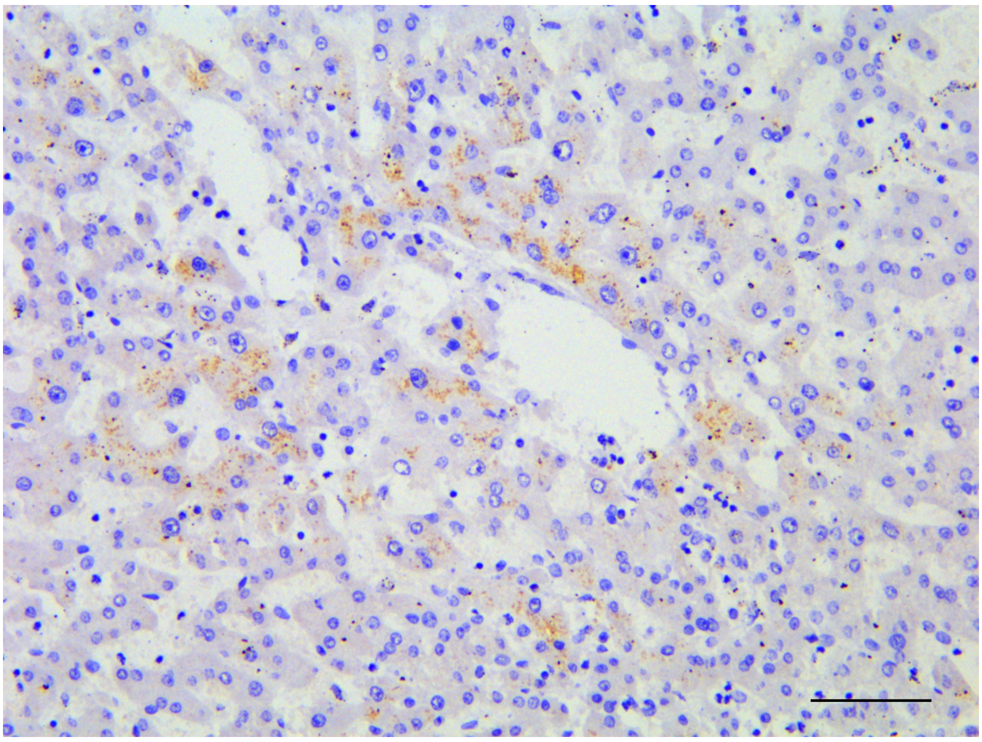

Supplemental figure 4. Representative images of liver immunohistochemistry staining for fH. To optimise our staining protocol for fH we utilised human liver tissue. The tissue was resected for possible metastatic disease and an area of unaffected tissue was used for staining. Staining for fH was evident in hepatocyes. fH − factor H. Bar represents 100 µm.
