# Supplementary material for: Progressive IgA Nephropathy Is Associated With Low Circulating Mannan-Binding Lectin–Associated Serine Protease-3 (MASP-3) and Increased Glomerular Factor H–Related Protein-5 (FHR5) Deposition
Source: Kidney Int Rep. 2017 Nov 29;3(2):426–38. doi: 10.1016/j.ekir.2017.11.015 (PMC5932138; doi:10.1016/j.ekir.2017.11.015)
Supplement: Figure S5 — Graphs showing the (a) correlation of plasma MASP-3 levels and urine protein-creatinine ratio (PCR) in patients (n = 223) with IgA nephropathy (IgAN); (b) the correlation of plasma M-ficolin with white cell count (WCC) in patients (n = 108) with IgAN; and (c) the correlation between mannan-binding lectin (MBL)-associated serine protease (MASP)-3 with alanine aminotransferase (ALT) in patients (n = 108) with IgAN. [file mmc5.pdf]

a

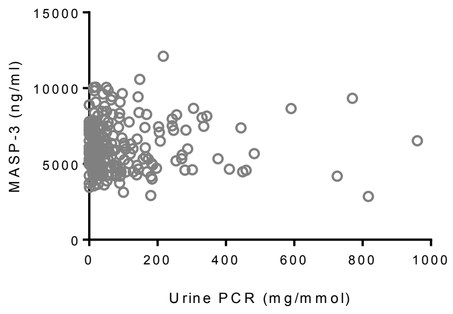

b

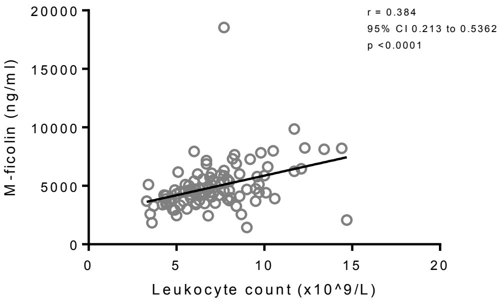

c

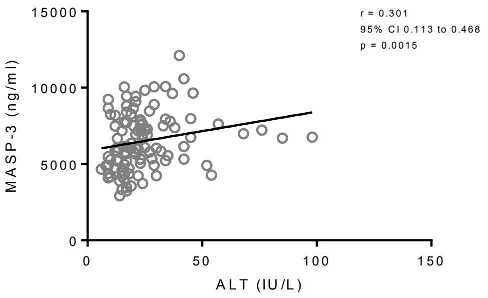

Supplemental figure 5. (a) Correlation of plasma MASP-3 levels and urine protein-creatinine ratio (PCR) in patients (n=223) with IgA nephropathy. (b) Correlation of plasma M-ficolin with white cell count (WCC) in patients (n=108) with IgA nephropathy. (c) Correlation between MBL-associated serine protease (MASP)-3 with alanine aminotransferase (ALT) in patients (n=108) with IgA nephropathy. *P* values derived from Spearman's rank correlation. CI – confidence interval.
