# Supplementary material for: Progressive IgA Nephropathy Is Associated With Low Circulating Mannan-Binding Lectin–Associated Serine Protease-3 (MASP-3) and Increased Glomerular Factor H–Related Protein-5 (FHR5) Deposition
Source: Kidney Int Rep. 2017 Nov 29;3(2):426–38. doi: 10.1016/j.ekir.2017.11.015 (PMC5932138; doi:10.1016/j.ekir.2017.11.015)
Supplement: Figure S6 — Graphs showing the correlation between estimated glomerular filtration rate (eGFR) and (a) M-ficolin, (b) mannan-binding lectin (MBL)-associated protein (MAp) 19, (c) MBL-associated serine protease (MASP)-3 levels, (d) L-ficolin, and (e) MASP-1 levels. [file mmc6.pdf]

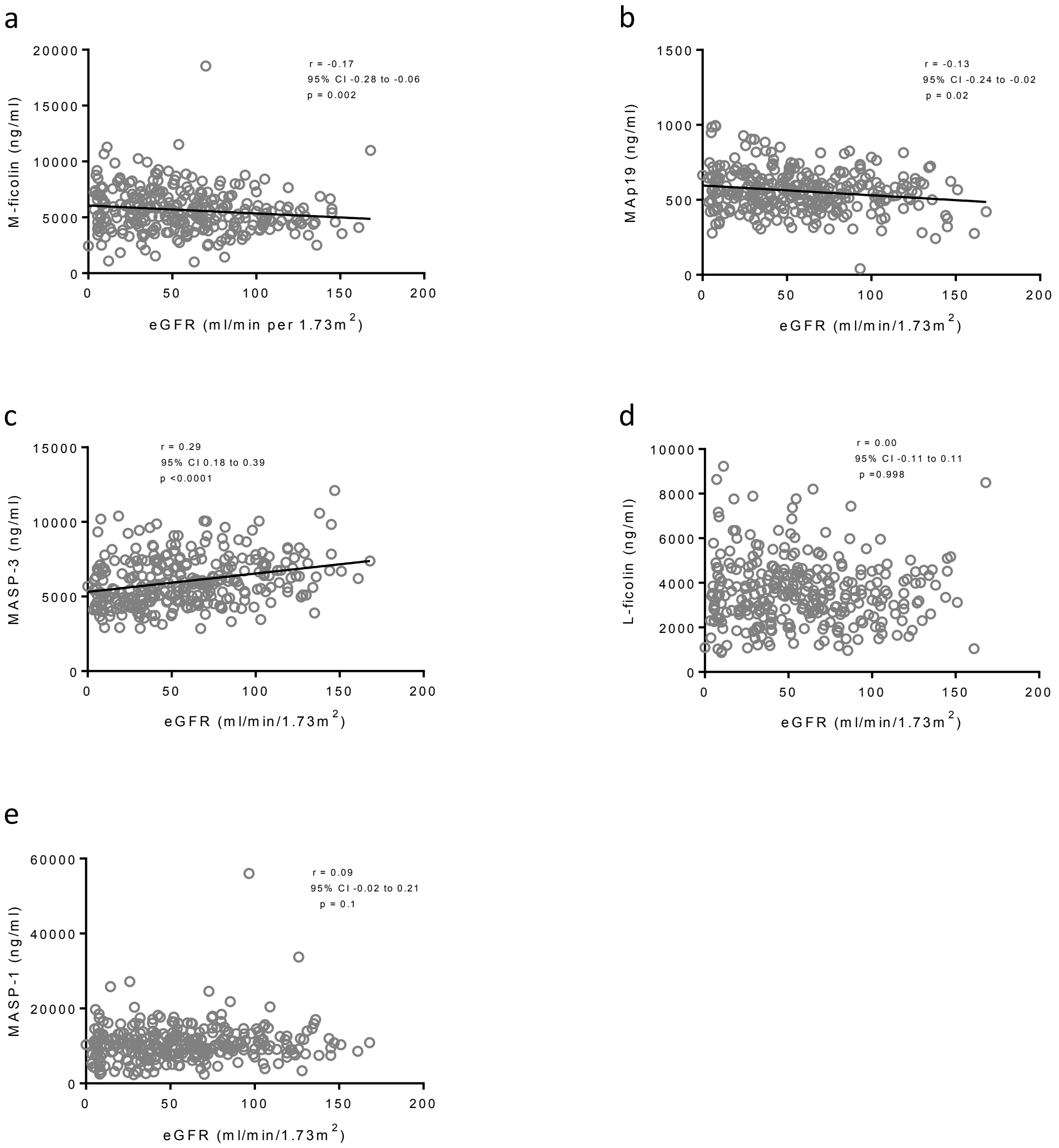

Supplemental figure 6. Correlation between estimated glomerular filtration rate (eGFR) and (a) M-ficolin, (b) MBL-associated protein (MAp) 19 , (c) MBL-associated serine protease (MASP)-3 levels, (d) L-ficolin, and (e) MASP-1 levels. *P* values derived from Spearman's rank correlation. CI – confidence interval.
