# Supplementary material for: Progressive IgA Nephropathy Is Associated With Low Circulating Mannan-Binding Lectin–Associated Serine Protease-3 (MASP-3) and Increased Glomerular Factor H–Related Protein-5 (FHR5) Deposition
Source: Kidney Int Rep. 2017 Nov 29;3(2):426–38. doi: 10.1016/j.ekir.2017.11.015 (PMC5932138; doi:10.1016/j.ekir.2017.11.015)
Supplement: Figure S7 — Table of circulating lectin pathway protein levels in stable and progressive IgA nephropathy. [file mmc7.pdf]

Lectin pathway protein	Progressive disease (n=191)	Stable disease (n=83)	Difference between medians	95% Confidence Interval
M-ficolin, ng/ml	5375 (1096-18538)	5335 (2954-11529)	40	-441 to 446
L-ficolin, ng/ml	3245 (872-9230)	3763 (1487-8500)	-518	-758 to 36
MASP-1, ng/ml	10013 (2439-24591)	10628 (3065-56002)	-615	-1660 to 184
MAp19, ng/ml	555 (242-996)	564 (305-818)	-9	-32 to 39

Supplemental figure 7. Lectin pathway protein levels in stable and progressive IgA nephropathy Vaules represent median with range of values in parentheses; MBL - mannan-binding lectin; MASP – MBL-associated serine protease; MAp, - MBL-associated protein.
