# Supplementary material for: Progressive IgA Nephropathy Is Associated With Low Circulating Mannan-Binding Lectin–Associated Serine Protease-3 (MASP-3) and Increased Glomerular Factor H–Related Protein-5 (FHR5) Deposition
Source: Kidney Int Rep. 2017 Nov 29;3(2):426–38. doi: 10.1016/j.ekir.2017.11.015 (PMC5932138; doi:10.1016/j.ekir.2017.11.015)
Supplement: Figure S8 — Renal immunohistochemistry staining for factor H–related protein 1 (FHR1) in IgA nephropathy. Representative images of tubular staining intensity 3+ (a), 2+ (b), 1+ (c), and 0 (d–f). (f) Representative image from a patient with complete FHR1 deficiency. Bar = 100 μm. Correlation of tubular FHR1 staining intensity with estimated glomerular filtration rate (eGFR, g) at the time of biopsy, urine protein-creatinine ratio (UPCR) at the time of biopsy (h), the presence (T1 or T2) or absence (T0) of tubular atrophy from the biopsy report (i); and disease severity at enrollment (j). No correlations reached statistical significance. [file mmc8.pdf]

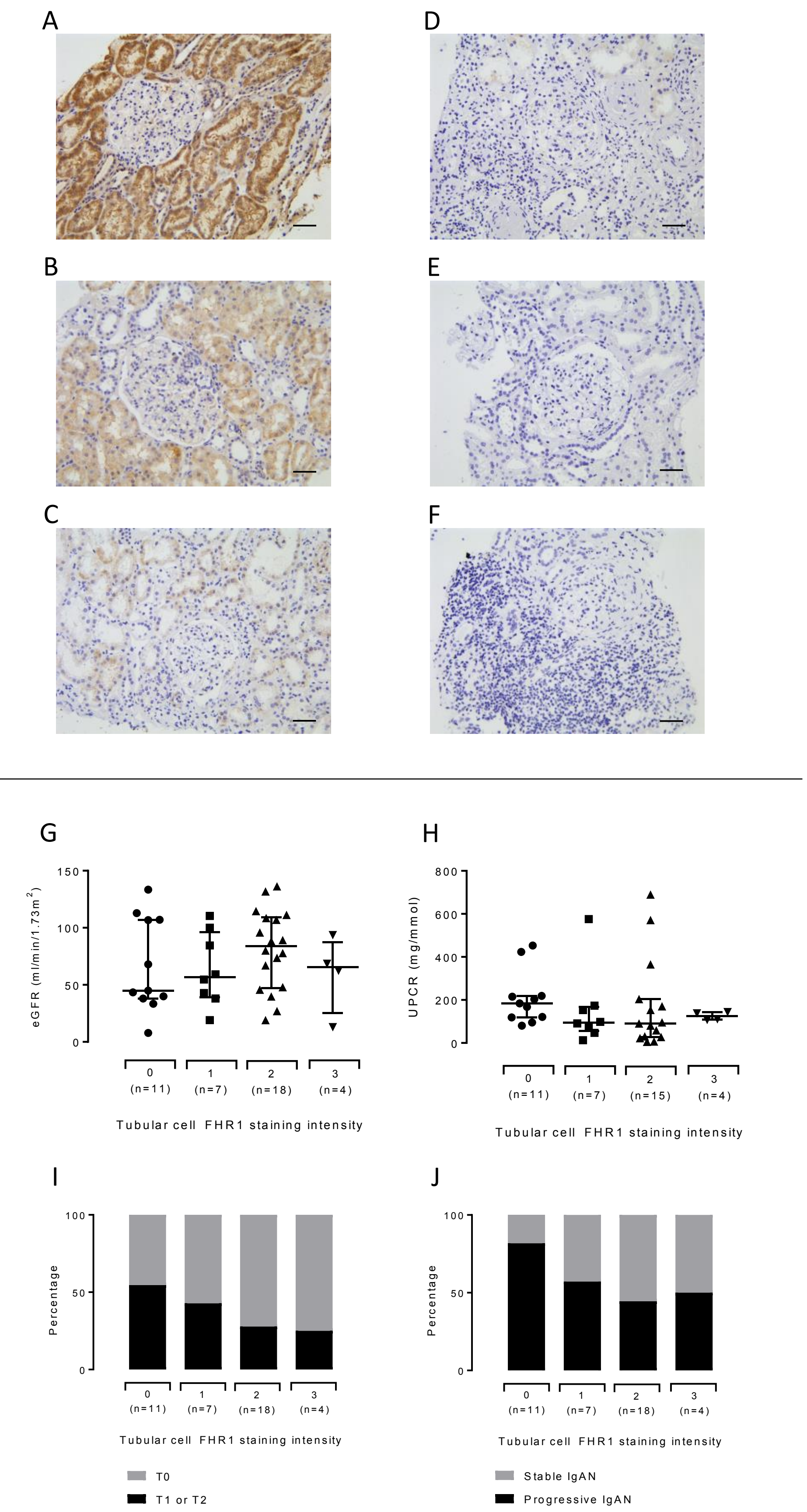

Supplemental figure 8. Renal immunohistochemistry staining for factor H-related protein 1 (FHR1) in IgA nephropathy. Representative images of tubular staining intensity 3+ (A), 2+ (B), 1+ (C), and 0 (D-F). F is a representative image from a patient with complete FHR1 deficiency. Bar represents 100 µm.

Correlation of tubular FHR1 staining intensity with estimated glomerular filtration rate (eGFR, G) at the time of biopsy, urine protein creatinine ratio (UPCR) at the time of biopsy (H), the presence (T1 or T2) or absence (T0) of tubular atrophy from the biopsy report (I); and disease severity at enrolment (J). No correlations reached statistical significance.
